# A phase II study of LFP therapy (5-FU (5-fluorourasil) continuous infusion (CVI) and Low-dose consecutive (Cisplatin) CDDP) in advanced biliary tract carcinoma

**DOI:** 10.1186/1471-2407-6-121

**Published:** 2006-05-06

**Authors:** Kazuma Kobayashi, Akihito Tsuji, Sojiro Morita, Tadashi Horimi, Tetsuhiko Shirasaka, Takashi Kanematsu

**Affiliations:** 1 Internal medicine, Clinical Oncology Group, Kochi Municipal Central Hospital, Kochi, Japan; 2 Radiology, Clinical Oncology Group, Kochi Municipal Central Hospital, Kochi, Japan; 3 Surgery, Clinical Oncology Group, Kochi Municipal Central Hospital, Kochi, Japan; 4 Department of Clinical Oncology, Kochi Health Science Center, Kochi, Japan; 5 Kitasato Institute for Life Sciences, Kitasato University, Tokyo, Japan; 6 Department of Transplantation and Digestive Surgery, Nagasaki University Graduate School of Biomedical Sciences, Nagasaki, Japan

## Abstract

**Background:**

Unresectable biliary tract carcinoma is known to demonstrate a poor prognosis. We conducted a single arm phase II study of LFP therapy (5-FU (5-fluorourasil) continuous infusion (CVI) and Low-dose consecutive (Cisplatin) CDDP) for advanced biliary tract malignancies basically on an outpatient basis.

**Methods:**

Between February 1996 and September 2003, 42 patients were enrolled in this trial.

**LFP therapy:**

By using a total implanted CV-catheter system, 5-FU (160 mg/m^2^/day) was continuously infused over 24 hours for 7 consecutive days and CDDP (6 mg/m^2^/day) was infused for 30 minutes twice a week as one cycle. The administration schedule consisted of 4 cycles as one course. RESIST criteria (Response evaluation criteria for solid tumors) and NCI-CTC (National Cancer Institute-Common Toxicity Criteria) (ver.3.0) were used for evaluation of this therapy. The median survival time (MST) and median time to treatment failure (TTF) were calculated by the Kaplan-Meier method.

**Results:**

Patients characteristics were: mean age 66.5(47–79): male 24 (54%): BDca (bile duct carcinoma) 27 GBca (Gallbladder carcinoma) 15: locally advanced 26, postoperative recurrence 16. The most common toxicity was anemia (26.2%). Neither any treatment related death nor grade 4 toxicity occurred. The median number of courses of LFP Therapy which patients could receive was two (1–14). All the patients are evaluable for effects with an over all response rates of 42.9% (95% confidence interval C.I.: 27.7–59.0) (0 CR, 18 PR, 13 NC, 11 PD). There was no significant difference regarding the anti tumor effects against both malignant neoplasms. Figure 2 Shows the BDca a longer MST and TTF than did GBca (234 vs 150, 117 vs 85, respectively), but neither difference was statistically significant.

The estimated MST and median TTF were 225 and 107 days, respectively. The BDca had a longer MST and TTF than GBca (234 vs 150, 117 vs 85, respectively), but neither difference was statistically significant.

**Conclusion:**

LFP therapy appears to be useful modality for the clinical management of advanced biliary tract malignancy.

## Background

Biliary tract cancers are rare in North America, with approximately 8,000 new cases diagnosed in 2003 [1]. However, bile duct carcinoma (BDca) and gallbladder carcinoma (GBca) are not rare in northern Japan [2], Taiwan [3], and South Korea [4]. In Japan, these malignancies are the sixth leading cause of cancer deaths, and in 1999, there were 8,557 deaths from BDca and 6,340 deaths from GBca [5]. As an surgical resection of the primary tumor and the areas of local extension remains the most effective therapy [6], even for non-curative operations [7]. However, in over 75% of the patients whose disease is locally advanced or already metastatic cases, the median survival time for patients receiving only the best possible supportive care is only about 6 months [1]. Furthermore, there is a high rate of both local and systemic recurrence, even after a curative resection [1,6]. As a result, an effective chemotherapy for biliary malignancy has been eagerly awaited. However, systemic single-agent chemotherapy has so far shown a poor efficacy [6,8], though many efforts has been done [9]. For example, the response rate of 5-fluorourasil (5-FU), cisplatin (CDDP), was 10–13%, and 8%, respectively [4], while new chemotherapeutic agents CPT-11, Gemsitabine, showed the poor response rate of 12.5% and 8%, respectively [3,4,10].

As a result, an effective combination chemotherapy has been eagerly anticipated. We spotlighted the combination of the two old anti-cancer agents, 5-FU, and CDDP.

In Japan, FP therapy combination of 5-FU continuous venous infusion (CVI) and low-dose consecutive CDDP (LFP) Therapy has been widely used since early 1990s for gastrointestinal advanced cancer [11,12]. Because of its low toxicity and relatively high response rate [13], LFP therapy has been widely used for the treatment of various unresectable advanced solid tumors, such as gastric cancer [14], hepatocellular carcinoma [15], pancreatic cancer [12], colon and head and neck [11]. Recent findings in experimental models have shown an additive or synergistic antitumor effects of LFP therapy. We observed that CDDP inhibited metionine transport into tumor cells, both in vitro and in vivo, with a synergic interaction by CDDP functioning as a modulator of 5-FU [11,12]. This synergistic effect was also associated with the induction of apoptosis [16] and the p53 pathway [17,18].

Based on these findings, we conducted a single arm phase II study of LFP therapy in patients with advanced BDca and GBca.

## Methods

### Eligibility criteria

This study protocol was approved by the Kochi Municipal Central Hospital, Japan and written informed consent was obtained from all the patients. The patients were required to have unresectable locally advanced or metastatic disease of the biliary-tract or gallbladder advanced carcinoma with measurable lesions on a computed tomography (CT) scan. Other eligibility criteria included an age 18 years or more, Eastern Cooperative Oncology Group (ECOG) performance status of 2 or less, estimated life expectancy of 12 weeks or more, adequate bone marrow function (leukocyte count > 3,500/μl, neutrophil count > 1,500/μl, and platelet count > 100,000/μl), bilirubin < 5.0 mg/dl, transaminases and alkaline phosphatase < 6 times upper limit of normal, and normal renal function tests (creatine level < 1.5 mg/dl, or creatinine clearance > 60 ml/min). No previous chemotherapy was permitted within 2 weeks. Radiotherapy and stenting to decompress the biliary tract was permitted. Patients with other clinically significant laboratory abnormalities, uncontrolled infection, concurrent severe medical problems unrelated to malignancy that would expose the patient to extreme risk, patients receiving another investigational drug within 30 days prior to study or receiving concurrent hormonal therapy, immunotherapy and those pregnant or lactating were excluded from the study. The study was conducted according to the Good Clinical Practices and the Declaration of Helsinki as amended in Hong Kong (1989).

### Treatment plans

All patients were admitted to the hospital for about 10 days for a pretreatment evaluation, the first cycle treatment, and observation for adverse effects. If the degree of toxicity was within Grade 0–2, a second cycle of treatment or more were continued on an outpatient basis.

A pretreatment evaluation included complete medical history, physical examination, evaluation of performance status, urinalysis, chest radiograph, and diagnostic studies assessment such as CT scan. When the patient meets the eligibility criteria, central venous catheter system with a heparin coated catheter (Anthron PU catheter; TORAY™ and a port (Celcite brachial; TORAY™ or Vital port mini; COOK™) is implanted according to the method of Hata et al [19] prior to treatment.

**Figure 1:**
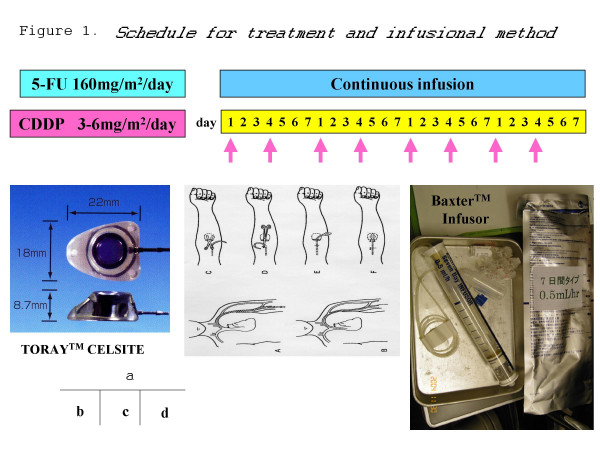
***Schedule for treatment and infusional method***. A schematic drawing of the chemotherapy schedule (a), and the Central Venous catheter system consists of PAS port (b), implantation technique (c) and portable infusion pump (d).

The treatment plan involved the administration of 5-FU (160 mg/m^2^/day) was continuously infused over 24 hours using a disposable infusion pump (7-day Infuser; Baxter™) and CDDP (3–6 mg/m^2^/day) diluted with normal serine was infused for half an hour. Hydration was not needed. These doses were determined based on our experience of the previous LFP therapy for hepatocellular carcinoma [15]. The administration schedule consisted of 5-FU for 7 consecutive days and CDDP twice a week (day 1 and day 4) for each of four weeks as one treatment course. The treatment schedule and CV catheter system was depicted on figure 1. Unless an exacerbation of the symptoms was observed, multiple courses of treatment were administered. When more than a grade 3 adverse effect was observed, a CDDP infusion was omitted and observed. If this omission was ineffective, 5-FU was also omitted. In case of hemoglobin < 8.0 g/dl, platelet count < 50,000/μl neutrophil count < 1,000/μl, blood transfusion of concentrated red blood cells (RBCs) or platelets, or granulocyte-stimulating factor (G-CSF) was applied.

We do not increase the dose of the anti-cancer agent during the chemotherapy protocol, even if the toxicity is low.

### Toxicity and response evaluation

Complete blood counts twice a week and biochemical examinations were weekly carried out. Toxicity was evaluated based on the National Cancer Institute Common Toxicity Criteria (NCI-CTC) ver.3. The response was classified based on the Response Evaluation Criteria in Solid Tumors Guidelines (RECIST criteria) [20], taking into account the measurement of the longest diameter only for all target lesions: complete response (CR)-the disappearance of all target lesions; partial response (PR)-at least a 30% decrease in the sum of the longest diameter of target lesions, taking as reference the baseline sum longest diameter; progressive disease (PD) -at least a 20% increase in the sum of the longest diameter of target lesions, taking as referenve the smallest sum longest diameter recorded since the treatment started or the appearance of one or more new lesions; no change (NC)-neither sufficient shrinkage to quality for partial response nor sufficient increase to qualify for progressive disease, taking as reference the smallest sum longest diameter since the treatment started. Patients with a CR, a PR, an NC, or a PD required a confirmatory disease assessment at least one month later. Target lesions were evaluated by a plain and enhanced CT scan and plain chest X-ray for each course.

### Statistical analyses

We used the Stat View J 5.0 software package (Abacus Concepts, Stat View. Abacus Concepts, Inc., Berkeley, CA, 1992–1998) for the statistical analysis. The time to Treatment Failure and the overall Survival Cumulative were obtained by the Kaplan-Meier method. The disease-free survival was compared by the Log rank test among the groups. Prognostic variables were evaluated by Cox's multivariate proportional hazard model. We defined the risk factors for LFP therapy for biliary tract malignancies in our study as significant factors based on both the Cox's and Kaplan-Meier's. A p value of less than 0.05 was considered to be statistically significant.

## Results

### Patient characteristics

From February 1996 to December 2003, 42 patients were enrolled into the present study, and all were evaluable for efficacy and toxicity analyses. They consisted of 24 males and 18 females. The mean age was 66.5(47–79). The number of patients with BDca and GBca were 27 and 15, respectively. Twenty-six patients who were initially diagnosed to have biliary tract malignancies were not eligible for surgery because of locally advanced disease and/or metastasis (locally advanced). Another 16 patients had local recurrence and/or distant metastasis after surgery (postoperative recurrence). Disease extension was such that 11 patients (BDca: 7, GBca: 4), had only primary or local recurrence patients had only metastatic disease (BDca: 9, GBca: 5), another 17 patients (BDca:11, GBca: 6) had both diseases. Four patients had previously undergone chemotherapy, including such treatments as CDDP + VP-16, 5-FU + mitomycin C + farmorubicin, adriamycin + 5-FU. Two of 4 patients experienced surgery before these chemotherapy (i.e. postoperative recurrence). Seven of BDca patients had palliative radiotherapy. Three of these seven underwent surgery before radiation (i.e. postoperative recurrence). There were some differences between BDca and GBca on age, and serum tumor marker levels (CEA and CA19-9), but neither was statistically significant. The patient characteristics are enumerated in Table 1.

### Response and time-to-event measures

The overall response rate (RR) was 42.9% (95% confidence interval C.I.: 27.7–59.0) with CR 0, PR 18, NC 13, PD 11, and clinical benefit was 73.8% (95% C.I.: 58.0–86.2). Patients with a PR, an NC or a PD required a confirmatory disease assessment at least two months later.

**Figure 2:**
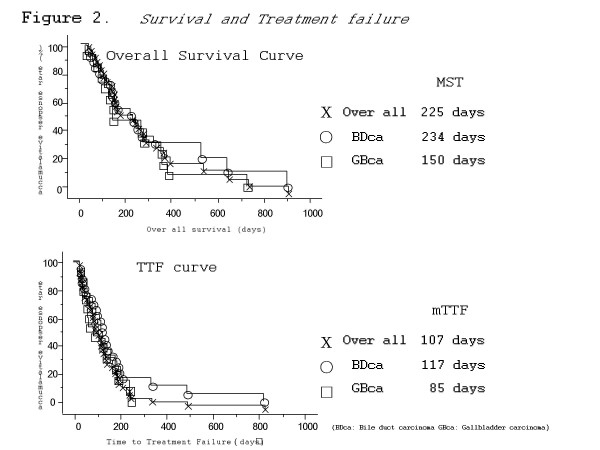
***Survival and Treatment failure***. Kaplan-Meier curves of Overall Survival (upper column) and Time to treatment failure (TTF) (lower column) are shown.

One GBca patient who got PR, could receive curative resection. The RR of primary lesion or locally advanced lesion was 50.0% (95% C.I.: 30.6–69.4), and the RR of metastatic lesions was 32.3%. Various RRs were demonstrated in Table 2. The responses of meatastatic lesions were as such; liver (CR:0, PR:6, NC:0, PD:3), lung (CR:0, PR:1 NC:3, PD:0), lymph node (CR:0, PR:3, NC: 6, PD2), miscellaneous (CR:0, PR: 2, NC: 2, PD: 1). There was no significant difference in terms of anti tumor effects against both malignant neoplasms. The overall MST was 225 days. The median TTF was 107 days. Figure 2 Shows the BDca a longer MST and TTF than did GBca (234 vs 150, 117 vs 85, respectively), but neither difference was statistically significant.

### Toxicity

As shown in Table 3, neither any treatment related death nor grade 4 toxicity occurred. Overall, the most common toxicity was anemia occurring in 26.2% of patients followed by nausea (19.0%). The most frequent grade 3 toxicity was appetite loss (14.3%). The occurrence of ascites and jaundice may be partly because of the outcome of the disease progression.

### Prognostic factors related survival and TTF

**Figure 3:**
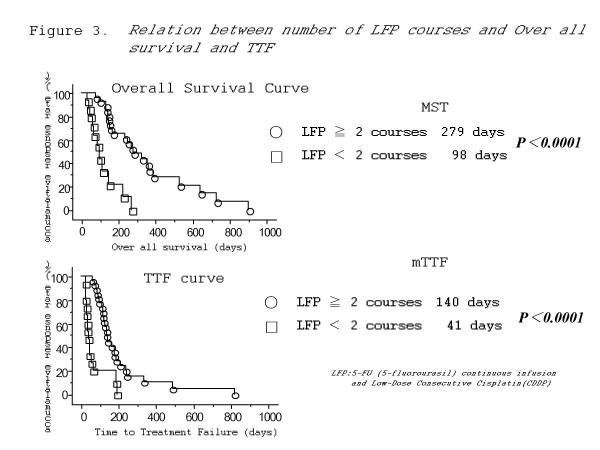
***Relation between number of LFP courses and Over all survival and TTF***. Differences by accomplished LFP (5-FU (5-fluorourasil) continuous infusion and Low-Dose Consecutive Cisplatin(CDDP)) courses are depicted regarding the overall Survival (upper column) and Time to treatment failure (TTF)(lower column).

An analysis of a Cox proportional hazard model showed that no significant factor was found prognostic factors for either the overall survival or TTF (Table 4). However, the patients with LFP courses ≧2 had both a longer overall survival and TTF than those with LFP courses < 2 as depicted in Figure 3. The distribution of the patients with LFP courses ≧2 was (PR/NC/PD = 16/8/3), while that of those with LFP courses ≤2 was (PR/NC/PD = 2/5/8).

### Cost benefit

The difference between the inpatient-basis and the outpatient-basis or home treatment of this therapy in respect to cost is shown in Table 5. This was calculated based on the assumption that one patient received one-month treatment of this LFP therapy covered by Japanese National Health Insurance. The cost for outpatient-basis/home LFP therapy was approximately equivalent to 996 U.S. dollars which was about one-sixth less than the cost on an inpatient-basis.

## Discussion

In the present study, we achieved an RR of 42.9% (95% C.I.: 27.7–59.0) with a median over all survival of 225 days and median TTF of 107 days. No grade 4 toxicity or treatment related death occurred. The cause of treatment failure of the other 37 patients was an aggravation of general condition due to primary disease, and not due to any adverse effect. LFP therapy showed a good compliance and the adverse effects were either tolerable or controllable.

The overall response rate of our study is relatively high for this type of tumor. However, our LFP method has achieved more than a 50% overall response rate in other tumors such as esophageal, gastric or colon cancers. Biliary tract cancer may be more malignant than other types of cancer.

One cycle of conventional LFP therapy consists of CDDP infusion consecutive five days per week and 24 hr continuous infusion of 5-FU consecutively 7 days per week [14]. Therefore, the patients were obliged to receive inpatient-basis treatment, which led to their inconvenience and a heavy burden due to the high admission fee. Regarding the five consecutive days of CDDP infusion, our preliminary study showed that CDDP infusion twice a week was sufficient to maintain the blood concentration of CDDP in order to achieve synergistic effect [15]. This fact and the application of a CV catheter system with PAS or vital port and portable infusion pump thus enable the patients to receive outpatient-basis treatment which is equivalent to the inpatient treatment in quality. As for central venous catheter, Knox reported six catheter infection cases occurred in 27 patients [6], but no complications related to the catheter system occurred in our study. Our good results were due to the easy technique of implantation associated with the catheter system [19]. In our hospital from July 1994 to December 2002, infection related to the CV-catheter system occurred in only 44 cases of total 1,350 implanted patients (3.4%).

We herein tried to compare our regimen with other combination chemotherapies [2,4-6,21-23] are summarized in Table 6. Our combination chemotherapy is thus considered to be effective enough to be recommended the biliary tract malignancy since our study achieved a low toxicity and high efficacy with a relatively higher RR and longer MST in comparison to these regimens. Kim's regimen [4] is also interesting since oral capesitabine was used. However, our results showed higher response rate and lower toxicity than Kim's.

One of the problems of our study was that the prognosis of the patients receiving less than two courses of LFP was remarkably poor as shown in Figure 3. Of these patients only one could receive second-line chemotherapy with CDDP/CPT-11. It is important to predict whether LFP therapy is effective or not. Fortunately, an effective method for predicting LFP therapy effectiveness for gastrointestinal cancers by detecting p53 has been reported [16-18]. This method may be applicable for biliary tract malignancy.

The present study used 5-FU continuous venous infusion (CVI) as an effector. If an oral drug which can help maintain a high blood concentration of 5-FU equivalent to or higher than that for the CVI-method exist, then the patients with biliary tract malignancy can avoid the need to use the catheter system but while still achieving an improved anti-tumor effect thus leading to an advanced quality of life. S-1 invented by one of the authors (T.S) [11] can thus be one of the candidates for this aim. S-1 is a novel oral fluoropyrimidine that consists of tegafur, which is a prodrug of 5-FU, 5-chloro-2, 4-dihydroxypyrimidine, which inhibits dihydropyrimidine dehydrogenase activity and potassium oxonate, which reduces gastrointestinal toxicity [11,24]. This feature helps to maintain a high blood concentration of 5-FU and less toxicity of digestive tract [11,24]. The result of 101 advanced gastric cancer patients with S-1 was reported to be 44.6% RR with 244 days of MST [25,26]. Furthermore, using the synergistic effect of LFP, the combination chemotherapy of CDDP and S-1 has also been performed for gastric cancer or pancreatic cancer at some institutes [13,25,27-30]. Many reports have so far described promising results. The application of low-dose CDDP and S-1 for biliary malignancies at our institute is now under consideration. Our study of LFP is thus considered to support the use of low-dose CDDP and S-1 regimen for BDca and GBca.

## Conclusion

In conclusion, this outpatient-basis LFP therapy is considered to be appropriate as a first-line treatment for either advanced or recurrent biliary tract cancer and it promises to help improve the quality of life of cancer patients while also facilitating the clinical management of such patients.

## Competing interests

The authors declares that they have no competing interests.

## Authors' contributions

KK carried out this study, and drafted this manuscript. AT conceived the design of present study. SM participated in assessing radiological findings. TH participated in its design and coordination. TS participated in the pharmacological basis of the study. TK participated in the coordination of this study and instructed the collaborators of this manuscript.

## Pre-publication history

The pre-publication history for this paper can be accessed here:



## Figures and Tables

**Table 1:** Patient Characteristics

Variables	Startification	Over all	BDca	GBca
Spieces of cancer		42	27	15
Sex	(male/female)	(24/18)	(17/10)	(7/8)
Age		66.5 ± 7.5	64.8 ± 8.2	69.5 ± 4.7
ECOG PS	(0/1/2)	(36/4/2)	(22/4/1)	(14/0/1)
Disease Status	(unresectable/postoperative recurrence)	(26/16)	(15/12)	(11/4)
Disease extension	locally advanced (primary)	26	15	11
	local recurrence	7	7	0
	liver metastasis	9	4	5
	lung metastasis	4	3	1
	lymphnode metastasis	11	4	7
	miscellaneous metastasis	5	5	0
Tumor marker	carcinoembryonic antigen (CEA)	16.7 ± 60.7	6.4 ± 17.2	36.7 ± 100.5
	CA19-9	3035.5 ± 9507.6	4495.3 ± 11688.8	407.9 ± 587.2
Previous chemotherapy	(yes/no)	(4/38)	(3/24)	(1/14)

Abbreveations

BDca: bile duct carcinoma, GBca: Gallbladder carcinoma, ECOG PS: Eastern Cooperative Oncology Group performance status

**Table 2:** Anti tumor effect

***Over all***						
	CR	PR	NC	PD	Response Rate (%)95%C.I. CR+PR/TOTAL	Clinical Response (%)95%C.I. CR+PR+NC/TOTAL

Over all	0	18	13	11	42.9 (27.7–59.0)	73.8 (58.0–86.2)
BDca	0	11	10	6	40.7 (22.4–61.2)	77.8 (57.8–91.4)
GBca	0	7	3	5	46.7 (21.1–73.5)	66.7 (38.4–88.2)

***Primary or local recurrence***						

	CR	PR	NC	PD	Response Rate (%)95%C.I. CR+PR/TOTAL	Clinical Response (%)95%C.I. CR+PR+NC/TOTAL

Over all	0	14	8	6	50.0 (30.6–69.4)	78.6 (59.0–91.7)
BDca	0	9	6	3	50.0 (26.0–74.0)	83.3 (58.6–96.5)
GBca	0	5	2	3	50.0 (18.6–81.4)	70.0 (34.7–93.5)

***Metastatic lesion***						

	CR	PR	NC	PD	Response Rate (%)95%C.I. CR+PR/TOTAL	Clinical Response (%)95%C.I. CR+PR+NC/TOTAL

Over all	0	10	12	9	32.3 (16.7–51.4)	71.0 (52.0–85.8)
BDca	0	6	9	5	30.0 (11.8–54.3)	75.0 (50.9–91.4)
GBca	0	4	3	4	36.4 (10.8–69.3)	66.7 (30.7–89.2)

Abbreveations

BDca: bile duct carcinoma, GBca: Gallbladder carcinoma

**Table 3:** Toxicity

Hematological Toxicity	Gr1	Gr2	Gr3	Gr4	≧Gr3 (%)	Over all (%)
anemia	0	11	0	0	0 (0)	11 (26.2)
leukopenia	0	1	0	0	1 (0)	1 (2.3)
thrombocytopenia	0	2	3	0	3 (7.1)	5 (11.9)
						
Non-hematological Toxicity	Gr1	Gr2	Gr3	Gr4	≧Gr3 (%)	Over all (%)

nausea	3	4	1	0	1 (2.3)	8 (19.0)
vomiting	1	1	3	0	1 (2.3)	5 (11.9)
appetite loss	0	0	6	0	6 (14.3)	6 (14.3)
oral mucositis	0	4	0	0	0 (0)	4 (9.5)
taste disturbance	0	1	0	0	1 (2.3)	1 (2.3)
upper GI tract bleeding	0	1	0	0	0 (0)	1 (2.3)
diarrhea	1	0	0	0	0 (0)	1 (2.3)
general fatigue	0	1	1	0	1 (2.3)	2 (4.7)
jaundice	0	0	4	0	4 (9.5)	4 (9.5)
serum AST/ALT level elevation	0	4	1	0	1 (2.3)	1 (2.3)
Serum creatinine level elevation	0	6	0	0	0 (0)	6 (14.3)

**Table 4:** Prognostic factors for over all survival and TTF

	Over all survival Time to treatment Failure					
	
Stratification	Hazard ratio	p-value	95% C.I.	Hazard ratio	p-value	95% C.I.
Sex (male/female)	0.123	0.0128	0.024–0.640	0.225	0.0095	0.073–0.695
Age (≧69/<69)	0.634	0.6082	0.111–3.692	2.380	0.2269	0.583–9.713
ECOG PS (PS0/PS1/PS2)	0.016	0.0015	0.001–0.259	0.230	0.0007	0.001–0.152
Species of tumor (BDca/GBca)	0.095	0.0198	0.013–0.688	0.001	0.0411	0.0856–0.943
Disease status (locally advanced/postoperative recurrence)	0.121	0.0179	0.021–0.695	0.802	0.7388	0.001–0.152
Radiation (yes/no)	8.369	0.1491	0.467–150.112	0.603	0.4586	0.158–2.299
Previous chemotherapy (yes/no)	0.143	0.0255	0.016–1.241	0.495	0.4086	0.094–2.622
Initial CEA level (≧1 UL/<1 UL)	1.098	0.9053	0.234–5.160	2.453	0.1267	0.775–7.763
Interval decreasing CEA level (yes/no)	0.224	0.2859	0.014–3.499	1.734	0.5968	0.260–11.568
Initial CA19-9 level (≧5 UL/<5 UL)	0.078	0.0255	0.014–3.499	1.734	0.5968	0.260–11.568
Interval decreasing CA19-9 level(yes/no)	1.449	0.7667	0.125–16.774	0.326	0.2600	0.047–2.290

(Cox proportional hazard model)

Abbreveations

BDca: bile duct carcinoma, GBca: Gallbladder carcinoma, ECOG PS: Eastern Cooperative Oncology Group performance status, CEA: carcinoembryonic antigen, UL: upper limit

**Table 5:** Cost-benefit

**Outpatient-basis or home**	**Inpatient-basis**
At home malignant tumor	Basic admission fee 472,900 yen
Administration fee 25,000 yen	Local infusion technical fee (addition to minute infusion) 46,200 yen
Portable infuser pump	
using fee 15,000 yen	Diet 63,600 yen
Medication	Medication
CDDP 20,000 yen	CDDP 20,000 yen
5-FU 15,000 yen	5-FU 15,000 yen
Tumor marker 5,000 yen	Tumor marker 5,000 yen
Other laboratory test 8,400 yen	Other laboratory test 8,400 yen
Imaging diagnosis 15,000 yen	Imaging diagnosis 15,000 yen
(antiemeric drug 16,000 yen)	(antiemeric drug 16,000 yen)
Total 103,600 (119,600) yen	Total 646,300 (662,300) yen

(One month treatment covered by Japanese National Health Insurance, A U.S. dollar is approximately equivalent to 104 Japanese yen)

**Table 6:** Current Combination Chemotherapy for Biliary tract cancer

Author (published year)	Number of Patients	Species of cancer	Regimen	RR (%)	MST (days)	mTTF (days)	Adverse effects (≧Gr3)	Treatment related death
Ishii(2004)	21	GBca	CEF(CDDP/5-FU/epirubicin)	33.3	177*	-	hematological toxicity (52.3%)	none
	25	GBca	FAM(5-FU/Doxorubicin/Mitomycin)	7.1		-	hematological toxicity (20%)	none
Lee (2004)	4	BDca	Gemsitabine/CDDP	50	270	150	thrombocytopenia (75%)	none
Doval(2004)	30	GBca	Gemsitabine/CDDP	36.6	140	126	nausea/vomiting (16%)	2
Malik(2003)	11	GBca	Gemsitabine/CDDP	64	294	196	anemia (45%)	none
Knox (2004)	27	BDca/CBca	Gemsitabine/5-FU	33	159	111	hematological toxicity (11%)	none
Malik(2003)	30	CBca	Leucovorin/5-FU	7.5	444	141	diarrhea (30%)	1
Kim (2003)	42	BDca/CBca	Capesitabine/CDDP	21.4	273	111	leucopenia (20%)	none

(* The result was overall survival of combined CEF and FAM)

Abbreveations

BDca: bile duct carcinoma, GBca: Gallbladder carcinoma, CDDP: Cisplatin, 5-FU: 5-fluorourasil, RR: response rate, MST: median survival time, mTTF: median time to treatment failure
